# Mechanisms of action and resistance to anti-HER2 antibody-drug conjugates in breast cancer

**DOI:** 10.20517/cdr.2024.06

**Published:** 2024-06-03

**Authors:** Khalil Saleh, Rita Khoury, Nadine Khalife, Claude Chahine, Rebecca Ibrahim, Zamzam Tikriti, Axel Le Cesne

**Affiliations:** ^1^International Department, Gustave Roussy Cancer Campus, Villejuif 94800, France.; ^2^Department of Head and Neck Oncology, Gustave Roussy Cancer Campus, Villejuif 94800, France.

**Keywords:** Trastuzumab, pertuzumab, trastuzumab emtansine, antibody-drug conjugate (ADC), trastuzumab deruxtecan, metastatic breast cancer, HER2, resistance

## Abstract

Human epidermal growth factor 2 (HER2)-positive breast cancer (BC) represents nearly 20% of all breast tumors. Historically, these patients had a high rate of relapse and dismal prognosis. The advent of HER2-targeting monoclonal antibodies such as trastuzumab followed by pertuzumab had improved the prognosis of HER2-positive metastatic BC. More recently, antibody-drug conjugates (ADCs) are now reshaping the treatment paradigm of solid tumors, especially breast cancer. Tratsuzumab emtansine (T-DM1) was one of the first ADC developed in oncology and was approved for the management of HER2-positive metastatic BC. In a head-to-head comparison, trastuzumab deruxtecan (T-DXd) defeated T-DM1 as a second-line treatment. The efficacy of ADCs is counterbalanced by the appearance of acquired resistance to these agents. In this paper, we summarize the mechanisms of action and resistance of T-DM1 and T-DXd, as well as their clinical efficacy. Additionally, we also discuss potential strategies for addressing resistance to ADC.

## INTRODUCTION

Breast cancer (BC) is the most common cancer in women worldwide and the second leading reason of cancer-related mortality. It accounts for 287,000 new diagnosed cases and approximately 43,000 deaths in the United States in 2022^[1]^. Human epidermal growth factor 2 (HER2)-positive BC represents nearly 20% of newly diagnosed patients. The HER2-neu gene encodes an oncoprotein that belongs to the epidermal growth factor receptor (EGFR) family consisting of four members^[2-4]^. This subtype of tumors has historically been linked to an aggressive phenotype, a higher rate of relapse, and a worse prognosis compared to other tumor subtypes if untreated^[5]^. This worse prognosis of HER2-positive BC has dramatically ameliorated with the development of anti-HER2 monoclonal antibodies (mAb). Trastuzumab, in combination with chemotherapy, was the first mAb approved in 1998 in HER2-positive metastatic BC^[6]^. Trastuzumab was also approved as a neoadjuvant and adjuvant treatment in combination with chemotherapy. Pertuzumab is another HER2-targeting recombinant humanized mAb. The combination of chemotherapy, trastuzumab, and pertuzumab represents the gold standard in first-line setting in metastatic HER2-positive BC based on the CLEOPATRA study^[7]^. The combination regimen has been then approved as a neoadjuvant treatment based on the NeoSphere phase II trial results^[8]^. Tyrosine kinase inhibitors (TKIs) targeting HER2, such as tucatinib, lapatinib, and neratinib, were also incorporated into the therapeutic arsenal of patients with metastatic BC^[9]^. The activity of all these drugs was limited by the development of resistance to HER2-targeting agents, and patients presented with disease progression. Antibody-drug conjugates (ADCs) are an emerging category of treatment developed in order to prevent or delay the emergence of resistance to treatment and to ameliorate efficacy in patients with metastatic BC. The first approved ADC was trastuzumab emtansine (T-DM1) as a second-line treatment in this subgroup of patients according to the randomized phase III EMILIA study in 2013^[10,11]^. Then came trastuzumab deruxtecan (T-DXd), a new-generation ADC, to replace T-DM1 in the second-line setting according to the results of a first randomized DESTINY-Breast03 phase III trial comparing T-DXd *vs*. T-DM1^[12,13]^. Table 1 summarizes the approvals of T-DM1 and T-DXd. Once again, the clinical efficacy of ADC is counterbalanced by the occurrence of acquired resistance. In this review, we focus on the structure of ADC, the mechanism of action and the clinical efficacy of T-DXd and T-DM1. We also discuss the mechanisms of resistance to these agents, and the strategies to overcome such resistance.

**Table 1:** Current approvals of T-DM1 and T-DXd

**Drug**	**Study name**	**Date of approval**	**Indication**
T-DM1	EMILIA	February 2013	HER2+ MBC previously treated with taxane and trastuzumab
T-DM1	KATHERINE	May 2019	Adjuvant treatment in HER2+ EBC with residual disease after NAC with taxane and trastuzumab
T-DXd	DESTINY-Breast01	December 2019	HER2+ MBC after two treatment lines
T-DXd	DESTINY-Gastric01	January 2021	HER2+ MGC or GEJ after a prior trastuzumab-based treatment
T-DXd	DESTINY-Breast03	May 2022	HER2+ MBC in second-line treatment
T-DXd	DESTINY-Breast04	August 2022	HER2-low MBC after prior systemic treatment
T-DXd	DESTINY-Lung02	August 2022	HER2-mutant metastatic NSCLC

T-DM1: Tratsuzumab emtansine; T-DXd: trastuzumab deruxtecan; MBC: metastatic breast cancer; EBC: early breast cancer; NAC: neoadjuvant chemotherapy; MGC: metastatic gastric cancer; GEJ: gastroesophageal junction; NSCLC: non-small cell lung cancer.

## STRUCTURE OF ADC

ADCs are composed of humanized mAbs binding to an antigen specific to the tumor and a payload (cytotoxic drug) via a linker that could be cleavable or non-cleavable^[14]^. The antibodies incorporated in ADCs are fully humanized and most commonly IgG1-based because of their easier fabrication and stronger immunogenic characteristics in comparison with other IgG subtypes^[15]^. The antibody contains two fragments that bind to antigens, known as Fabs, and a constant fragment (Fc). Fabs mediate the identification of the antigen, while Fc is responsible for the interaction between the antibody and the effector immune cells^[16]^. An optimal antibody might present high affinity of binding to the correspondent target with low immunogenicity and good internalization with a relatively long plasma half-life^[17]^. Activation of immune cells, activation of the complement complexes, induction of complement-dependent cytotoxicity (CDC), activation of antibody-dependent cell-mediated cytotoxicity (ADCC), and antibody-dependent cell-mediated phagocytosis (ADCP) represent the main immune effector function of the antibody^[18]^.

The payload (cytotoxic drug) is very potent and represents the most important element of the ADC. Cytotoxic payloads are divided into several categories such as anti-tubulin (maystatine analogs, auristatin analogs), topoisomerase I inhibitors (deruxtecan, govitecan), DNA-damaging agents (calicheamicins, duocarmazine, pyrrolobenzodiazepines), and RNA polymerase II inhibitor (amanitin). These payloads had an unfavorable therapeutic index if given intravenously as non-conjugated drugs^[19]^.

The antibodies are connected to the cytotoxic payloads via linkers that lead to the stabilization of the ADCs and affect the pharmacokinetic characteristics of these drugs. These linkers might be cleavable, a process linked to factors specific to the tumors, such as pH changes (acid pH), reduction-oxidation conditions, or lysosomal enzymes. They can also be non-cleavable and require more processing to release cytotoxic payloads. In the case of non-cleavable linkers, the whole proteolytic degradation of the antibody happens in the lysosomes, a mechanism that might influence the payload. However, cleavable linkers presented lower plasma stability and shorter half-lives compared to non-cleavable linkers^[20-22]^.

Drug-to-antibody ratio (DAR) is an important indicator and characteristic of ADCs. It refers to the number of drug molecules linked to the antibody. In general, the potency of ADCs is positively correlated to DAR. High DAR could be associated with quick clearance of the drug and, probably, higher toxicity. In contrast, lower DAR could be associated with decreased activity of ADC and increased therapeutic index^[23,24]^.

## MECHANISM OF ACTION AND CLINICAL ACTIVITY

### T-DM1

As previously mentioned, T-DM1 is the first anti-HER2 ADC approved for the management of metastatic BC. It is composed of trastuzumab as HER2-targeting IgG1 monoclonal antibody and linked to emtansine (also known as DM1), a microtubule-inhibitory agent derivative of maytansine, via a non-cleavable linker. T-DM1 has a DAR of 3.5^[21]^. The mechanism of action of T-DM1 is complex. It is based on selective liberation of emtansine to HER2-positive tumor cells, inhibition of HER2 signaling by the trastuzumab, and shedding of the HER2 extracellular domain. T-DM1 can also evoke ADCC, similar to trastuzumab^[25-27]^. Another mechanism of action of T-DM1 that has been reported by Hunter *et al*. was the mitotic catastrophe that inhibits tumor growth with prolonged treatment of HER2-positive xenografts with T-DM1. It is characterized by the presence of a high proportion of tumor cells with aberrant mitotic figures^[27]^. The clinical activity of T-DM1 was evaluated in the EMILIA phase III study. It showed the superiority of T-DM1 over capecitabine combined with lapatinib in second-line treatment. T-DM1 had statistically significant higher median PFS (9.6 *vs*. 6.4 months; *P* < 0.001) and median OS (30.9 *vs*. 25.1 months; *P* < 0.001). In 2013, the FDA approved T-DM1 as a second-line treatment for the management of patients with HER2-positive metastatic BC, according to the results of this study^[10,11]^. However, T-DM1 failed to be positioned as a first-line treatment in the MARIANNE phase III study that randomized T-DM1 alone or with pertuzumab *vs*. taxane and trastuzumab. T-DM1 was not superior to control group^[28]^. Moreover, the incorporation of T-DM1 in the neoadjuvant treatment was not successful in the KRISTINE trial that compared T-DM1 in combination with pertuzumab *vs*. docetaxel, carboplatin, trastuzumab, and pertuzumab^[29,30]^. More recently, T-DM1 has been positioned in adjuvant treatment in patients who had remaining residual disease following anti-HER2-based neoadjuvant treatment based on the results of the KATHERINE phase III study. T-DM1 significantly reduced the risk of relapse or death by 50% in comparison with trastuzumab alone^[31]^. In this trial, the most frequent adverse events (AEs) contributing to T-DM1 discontinuation were thrombocytopenia (4.2%), increased bilirubin level (2.6%), and transaminase elevation (1.6%). The benefit of adjuvant T-DM1 is demonstrated in this subgroup of patients despite liver toxicity leading to treatment discontinuation in 1% to 2% of patients^[31]^.

### T-DXd, DS8201

T-DXd is a third-generation ADC composed of trastuzumab, linked to deruxtecan, a potent topoisomerase I inhibitor and is an exatecan derivantive, via a cleavable linker^[32]^. T-DXd is characterized by multiple features such as its potent payload, short systemic half-life avoiding systemic exposure, a DAR of 8, and the bystander effect^[33]^. The phase II DESTINY-Breast01 clinical trial investigated the dosage of 5.4 mg/kg of T-DXd every three weeks, in pretreated patients with HER2-positive metastatic BC and resistant to T-DM1. The clinical activity of T-DXd was confirmed with an ORR of 61% and the median PFS was 19.4 months. Interstitial lung disease (ILD) was reported in nearly 15% of patients and two patients died^[34]^. Based on these results, the FDA and the EMA approved T-DXd for the treatment of patients with HER2-positive metastatic BC who had received two or more anti-HER2-based regimens including T-DM1. More recently, the DESTINY-Breast02 confirmed the superiority of T-DXd over trastuzumab or lapatinib in combination capecitabine in patients with HER2-positive metastatic BC who had experienced treatment failure with T-DM1, with no additional safety concerns. The median PFS was 17.8 months in the T-DXd group *vs*. 6.9 months in the control arm (*P* < 0.0001)^[35]^. Ultimately, T-DXd was directly compared with T-DM1 in the DESTINY-Breast03 phase III in patients with metastatic BC previously treated with taxane and trastuzumab. The study met its primary endpoint of PFS. In this setting, T-DXd dethroned T-DM1 with impressive results in terms of PFS and OS (that was a secondary endpoint). The median PFS was 28.8 months with T-DXd *vs*. 6.8 months for T-DM1 patients (*P* < 0.0001). The median OS was not reached in both arms at a median follow-up of nearly 2 years with a HR of 0.64 (*P* = 0.0037) in favor of T-DXd^[12,13]^. Once again, the most frequent grade 3 or more treatment-related adverse events (TRAEs) were neutropenia (19.1%) and thrombocytopenia (7%). ILD was reported in 10.5% of patients, with no death occurring. Based on these results, T-DXd is positioned as standard second-line treatment in patients with metastatic BC, pushing T-DM1 to later treatment options and sparking debate on its role in subsequent lines. Moreover, T-DXd was also evaluated in patients with HER2-low metastatic BC who failed at least one previous line of chemotherapy in the DESTINY-Breast04 phase III trial in comparison to the physician’s choice of chemotherapy. The trial met its primary endpoint of PFS in the hormone receptor-positive cohort. The median PFS was 10.1 months in the T-DXd arm *vs*. 5.4 months in the control arm (*P* < 0.001). The median OS was 23.9 months in the T-DXd arm *vs*. 17.5 months in the control group (*P* = 0.003). No new safety concerns were reported^[36]^. This trial led to the approval by the FDA of T-DXd for the treatment of adult patients with metastatic HER2-low breast cancer who failed at least one line of chemotherapy. The efficacy of T-DXd in patients with metastatic HER2+ BC with active brain metastases (BMs) has been reported in the TUXEDO-1 phase II trial. T-DXd was associated with an intracranial ORR of 73% (11/15) (95%CI: 48%-89%), including a 13% complete response (CR)^[37]^. The DEBBRAH phase II trial also revealed an intracranial ORR of 46.2% in patients with HER2+ metastatic BC with active BMs^[38]^. Moreover, subgroup analysis of patients with stable BMs included in the DESTINY-Breast01 and DESTINY-Breast03 trials reported interesting rates of intracranial ORR [58.3 (95%CI: 36.3%-77.9%) and 67.4%, respectively]^[39,40]^. Table 2 summarizes major ongoing clinical trials of T-DXd as monotherapy.

**Table 2:** Major ongoing trials as monotherapy in BC

**Study**	**Agents**	**Phase**	**No. of patients**	**Patients**	**Primary endpoint**
NCT05113251 (DESTINY-Breast11)	ddAC THP *vs*. T-DXd or T-DXd followed by THP	III	900	High-risk HER2+ early-stage BC	Rate of pCR
NCT05704829 (ADAPTHER2-IV)	T-DXd *vs*. CT + T + P	II	402	Neoadjuvant in low-intermediate risk HER2+ BC	Rate of pCR dDFS
NCT05900206 (ARIADNE)	T-DXd *vs*. standard of care	II	370	Neoadjuvant treatment HER2+ BC	Rate of pCR
NCT04622319 (DESTINY-Breast05)	T-DXd *vs*. T-DM1	III	1,600	High-risk HER2+ BC with residual disease in breast or axillary lymph nodes following neoadjuvant therapy	IDFS
NCT04784715 (DESTINY-Breast09)	T-DXd *vs*. T-DXd + P *vs*. THP	III	1,157	HER2+ first-line MBC	PFS
NCT04739761 (DESTINY-Breast12)	T-DXd	IIIb/IV	506	Previously treated HER2+ MBC with or without BM	ORR PFS
NCT05950945 (DESTINY-Breast15)	T-DXd	IIIb	250	MBC HER2-low or HER2 IHC 0	TTNT
NCT05744375 (TRANSCENDER)	T-DXd	II	41	First-line treatment MBC resistant to T + P	ORR
NCT06048718 (TUXEDO-4)	T-DXd	II	27	HER2-low MBC with newly diagnosed or progressing BM	ORR

BC: Breast cancer; THP: taxane + Herceptin + pertuzumab; T: trastuzumab; P: pertuzumab; T-DXd: trastuzumab deruxtecan; CT: chemotherapy; pCR: pathological complete response; dDFS: distant disease-free survival; T-DM1: tratsuzumab emtansine; IDFS: invasive disease-free survival; MBC: metastatic breast cancer; PFS: progression-free survival; BM: brain metastasis; ORR: objective response rate; TTNT: time to next treatment.

## MECHANISMS OF RESISTANCE TO ANTI-HER2 ADC

Due to the complexity of the structure and mechanism of action of ADCs, which involve multiple processes, resistance can occur at any stage. It could be related to the expression of an antigen and its identification, or to internalization of the drug and its degradation, or to the release of payload and finally regulation of the apoptosis. To be noted that the large part of data discussed below had been obtained in preclinical models.

### Antigen-related resistance

The most important mechanisms of resistance to HER2-targeting ADCs are related to the exposed antigen. After a long exposure to treatment, the level of expression of the receptor may decrease or structural alterations can occur. After a long treatment with T-DM1 and a high selective pressure, cells showing increased HER2 expression are cleared while those showing decreased HER2 receptor expression are subjected to clonal evolution^[41]^. Loganzo *et al*. reported the results of an *in vitro* experience of trastuzumab-maytansinoid (TM-ADC), which is an ADC similar to T-DM1, and BC cells. Two different lines of cells were used: MBA MB-361-DYT2 parental cells, and JIMT1 tumoral cells resistant to first-line treatment with trastuzumab. JIMT1 cells exhibited resistance to TM-ADC and presented responses to other chemotherapies that include anti-tubulin, demonstrating that decreased HER2 expression after months of selective pressure could contribute to drug resistance^[42]^.

Moreover, the role of tumor heterogeneity in antigen expression and efficacy of ADCs was also reported in the KRISTINE trial. The presence of HER2-negative area in 10% of the cases by FISH or the detection of ERBB2 amplification between 5% and 50% of tumor cells defined HER2 heterogeneity. The pathologic complete response rate was 0% in the 10% of patients presenting tumor heterogeneity^[43]^. Furthermore, in the KRISTINE trial, patients with high levels of HER2 heterogeneity before T-DM1 had lower survival outcomes in comparison with those with low heterogeneity^[43]^. Similar results were found in the ZEPHIT trial that showed shorter times to treatment failure in patients with high levels of HER2 heterogeneity on HER2-PET CT scans^[44]^. Additionally, the antigen ectodomain may present truncated forms of HER2. It was reported as one of the potential resistance mechanisms but has not yet been proven in ADC. It has been initially proven with trastuzumab; tumors with truncated P95HER2 exhibited resistance to trastuzumab, whereas tumors with normal receptors were responsive to trastuzumab^[45]^. Heregulin, also known as neuregulin (NRG), which is a ligand for ERBB3 and ERBB4 receptors, could be another potential mechanism that might modulate the sensitivity to ADCs. NRG is a member of the epidermal growth factor (EGF) group, altering the sensitivity to T-DM1 and stimulating the heterodimerization of HER2 with HER3 and HER4^[46]^. Moreover, it seems that HER3 overexpression might be associated with resistance to HER2-targeting drugs such as T-DM1 and trastuzumab^[47]^. The inhibition of HER3 reversed acquired resistance to trastuzumab in *in vitro* xenografts derived from HER2-overexpressed gastric cancer^[48]^.

### Payload-related resistance

Another suggested mechanism of resistance to ADC is payload-related resistance that was initially observed in patients with non-Hodgkin lymphomas^[49]^. Takegawa *et al*. demonstrated that cells with resistance to T-DM1 had normal overexpression of HER2 but presented upregulated ABCC2 and ABCG2 expression, two ABC transporters that play an important role in chemotherapy resistance. Interestingly, T-DM1 efficacy was restored with the inhibition of these transporters, suggesting that amplified DM1 efflux contributed to resistance to T-DM1^[50]^.

#### Intrinsic mechanisms of resistance to ADCs

One of the intrinsic mechanisms of resistance is the failure of internalization of HER2 by endocytosis. Numerous pathways of internalization were reported, such as clathrin-mediated endocytosis mediated by clathrin, the most common way of endocytosis of ADCs, endocytosis mediated by caveolae, and endocytosis independent of clathrin-caveolin^[51]^. Sung *et al*. generated multiple in-vitro models of resistance to T-DM1^[52]^. The aforementioned mechanism (reduced HER2 expression) was found in two models. However, N87-TM model had normal transporters’ expressions and the same level of HER2 expression. N87-TM resistant cells to T-DM1 internalized HER2 receptor via caveolin-1 (CAV-1)-coated vesicles. In fact, the overexpression of CAV-1 was negatively correlated with HER2 expression over cell membranes and was associated with decreased colocalization of lysosomes and reduced T-DM1 sensitivity. Preclinical data demonstrated that colocalization of CAV-1 and T-DM1 was associated with lower sensitivity to T-DM1, suggesting CAV-1 as a predictive biomarker of T-DM1 resistance and other types of ADCs with non-cleavable linkers^[52-55]^. Moreover, endophilin A2, a protein involved in clathrin-independent endocytosis and encoded by SH3GL1, increases HER2 internalization in patients with HER2-positive BC and is associated with a higher response of tumor cells to T-DM1 and trastuzumab. It has also been demonstrated that the inhibition of SH3GL1 in cancer cells reduced the internalization and contributed to lower cytotoxicity mediated by T-DM1^[56]^.

After the internalization of ADCs and their target molecules via endocytosis, they are driven to lysosomes, and cytotoxic agents are released after chemical and enzymatic cleavage. An impaired lysosomal function can contribute to resistance to ADCs. In fact, Ríos-Luci *et al*. isolated three resistant HER2-positive clones following treatment with T-DM1. These clones had the same expression of HER2, normal internalization and trafficking pathways. However, they presented increased pH levels in the lysosomes, along with altered proteolysis, resulting in the accumulation of T-DM1 and subsequently limiting its antitumor activity^[57]^. Moreover, defective liberation of antineoplastic drugs from lysozymes or defective traffic to cytoplasm might be implicated in the resistance to ADCs. *In vitro* models showed that the silencing or loss of SLC46A3, a gene encoding for a protein that is a direct transporter of maytansine-based catabolites outside the lysosomes, led to the accumulation of catabolites in the lysosomes and subsequent acquired T-DM1 resistance^[58]^. Regarding T-DXd, the DAISY multicenter phase II trial was the first study investigating the mechanisms of action of T-DXd as well as the mechanism of resistance that was unclear. The DAISY trial enrolled patients with metastatic BC into three cohorts: HER2-positive cohort (*n* = 72 patients), HER2-low (*n* = 74 patients), and HER2-zero (*n* = 40 patients). T-DXd was given at the dosage of 5.4 mg/kg every three weeks until progression or unacceptable toxicity. The primary endpoint of the study was the best objective response. Interestingly, tumor samples were required before the beginning of the treatment and at progression. In addition, whole exome sequencing was performed on these biopsies. It has been demonstrated that T-DXd distribution was lower in the HER2-zero cohort in comparison with the HER2-positive cohort (*P* = 0.05). Additionally, enrichment of serotonin and G-protein coupled receptor signaling was found in the HER2-positive cohort after T-DXd treatment. The WES found that mutations in SLX4, a gene that plays an important role in regulating endonucleases and DNA damage repair, were present in 20% of patients. The DAISY trial found that depletion of SLX4 led to cancer cell resistance to treatment. Moreover, loss-of-function mutations could be identified as a potential mechanism of resistance to treatment^[59]^.

Drug efflux pumps were also reported as a mechanism of resistance to treatment by enhancing treatment ejection from tumor cells and could contribute to the occurrence of cancers with multidrug-resistant (MDR) phenotypes^[60]^. It could also be considered as a mechanism of resistance to ADC. The most frequent phenotype is the MDR1-mediated efflux. For example, maytansinoids are substrates of ABC transporters such as MDR1^[61]^. Moreover, *in vitro* data showed that the inhibition of MDR1 led to the reversal of the resistance to T-DM1^[62]^.

### Signaling pathways activation

Another potential mechanism that might contribute to resistance to ADCs is the activation of signaling pathways. The most common pathway involved is the PI3k/AKT/mTOR pathway, which is responsible for tumor growth, metabolism, and survival. It has been reported that the activation of the PI3K pathway can contribute to reduced response to ADCs, decreased activity of the cytotoxic drug, and longer survival of the cells in patients treated with trastuzumab who have PTEN deletions or PIK3CA mutations^[63]^. To date, available data from the EMILIA trial show that patients with PIK3CA mutation or PTEN deletion and receiving T-DM1 had the same survival outcomes compared to those without genetic alterations^[64]^. Moreover, Wu *et al*. reported that the overexpression of Wnt3 favors higher expression of β-catenin, increased cell proliferation rate, cell invasiveness and resistance to trastuzumab, indicating that the activation of the Wnt/β-catenin pathway might be involved in resistance to ADCs^[65]^. Another potential mechanism of resistance is the overexpression of the trophoblast cell-surface antigen 2 (TROP-2), which is a transmembrane glycoprotein that could be implicated in the akt phosphorylation in different tumoral cells, and in the activation of several signaling pathways such as PI3K/Akt, MAPK/ERK, and Wnt/ β-catenin pathways^[66]^. Recently, Gion *et al*. reported a retrospective analysis from the PHERGain trial of the relationship between TROP-2 expression and pathological complete response (pCR). Patients with TROP-2 expression had significantly lower pCR (*P* = 0.01). These findings suggest that TROP-2 could be a potential biomarker of resistance^[67]^. Moreover, the Topoisomerase II Alpha (TOP2A) gene is located at chromosome 17 and near the HER2/neu gene. It encodes for topoisomerase II alpha protein. Interestingly, it has been reported that 35% of patients with BC harboring HER2 amplification present with co-amplification of the TOP2A gene^[68]^. The topoisomerase II alpha protein is the target of anthracyclines, notably doxorubicin and epirubicin^[69]^. The role of TOP2A in the resistance to anti-HER2 targeted therapies is unclear. However, it could be intriguing to develop agents that target HER2 and TOP2A.

## OVERCOMING RESISTANCE TO ADC

Multiple strategies are currently under evaluation in order to prevent or reverse resistance to anti-HER2 ADCs that remains a significant challenge, and to ameliorate their clinical activity in metastatic BC. These strategies might be the use of other ADCs, more specifically, next-generation ADCs with different target or payload or DAR, or combination with other agents such as tyrosine kinase inhibitors (TKIs), immune checkpoint inhibitors (ICIs), and synthetic DNA-damaging agents. Tables 3 and 4 summarize major ongoing clinical trials evaluating T-DM1 and T-DXd combined with other drugs. Moreover, Durbin *et al*. reported in preclinical models that the reduction of antigen/antibody complex recycling to the membrane could be a possible target for pharmacological intervention^[70]^.

**Table 3:** Ongoing studies evaluating T-DM1 combined with other drugs in BC

**Trial**	**Agents**	**Phase**	**Number of patients**	**Patients**	**Primary endpoint**
NCT05673928 (TUCATEMEB)	Tucatinib in combination with T-DM1	II	30	Metastatic HER2-positive cancers	Intracranial antitumor activity
NCT04873362 (Astefania)	T-DM1 + placebo or atezolizumab	III	1,700	HER2-positive BC with high risk of relapse in the adjuvant setting	IDFS
NCT04457596 (CompassHER2 RD)	T-DM1 + placebo or tucatinib	III	1,031	HER2-positive BC with residual disease following neoadjuvant HER2-based treatment	IDFS
NCT05560308	T-DM1 + pyrotinib maleate	II	50	HER2+ MBC after progression on TKI	ORR

T-DM1: Tratsuzumab emtansine; BC: breast cancer; IDFS: invasive disease-free survival; MBC: metastatic breast cancer; TKI: tyrosine kinase inhibitor; ORR: overall response rate.

**Table 4:** Ongoing studies evaluating T-DXd associated with other agents in BC

**Study**	**Agents**	**Phase**	**Number of patients**	**Patients**	**Primary endpoint**
NCT04539938 (HER2CLIMB-04)	Tucatinib + T-DXd	II	70	HER2-positive MBC progressing on trastuzumab + taxanes	ORR
NCT05633979	T-DXd + valemetostat	Ib	37	HER2-low/0 MBC	Safety MTD ORR RDE
NCT05795101 (TRUDI)	T-DXd + durvalumab	II	63	Previously untreated with HER2+/low Inflammatory BC	pCR
NCT05372614	T-DXd + neratinib	I	18	Metastatic HER2-altered tumors	DLTs TEAEs
NCT04538742 (DESTINY-Breast07)	T-DXd + durvalumab or tucatinib or pertuzumab or paclitaxel	I/II	245	Previously untreated HER2-positive MBC	AEs SAEs
NCT04042701	pembrolizumab + T-DXd	I	115	HER2-positive tumors after progression on T-DM1 or HER2-low MBC	DLTs ORR
NCT04585958	Olaparib + T-DXd	I	55	Metastatic HER2-altered tumors	MTD, RP2D AEs

T-DXd: Trastuzumab deruxtecan; BC: breast cancer; MBC: metastatic breast cancer; ORR: objective response rate; MTD: maximum tolerated dose; RDE: recommended dose for expansion; pCR: pathologic complete response; DLTs: dose-limiting toxicities; TEAEs: treatment-emergent adverse events; AEs: adverse events; SAEs: serious adverse events; T-DM1: tratsuzumab emtansine; RP2D: recommended phase 2 dose; AEs: adverse events.

### Next-generation ADCs

Regarding next-generation ADCs, the previously discussed DESTINY-Breast02 phase III trial is the first randomized study proving that an ADC (T-DXd) can overcome the resistance to another one (T-DM1)^[35]^.

Trastuzumab duocarmazine, formerly named SYD985, is a HER2-targeting monoclonal antibody coupled to a duocarmycin (alkylating agent) via a C linker. The DAR of SYD985 is 2.6. It has been investigated in a phase I study enrolling patients with locally advanced or metastatic solid tumors with HER2 immunohistochemistry of 1+ or more. The study included patients with heavily pretreated metastatic BC. The dose-limiting toxicity was 2.4 mg/kg (related to pneumonitis) and the recommended phase 2 dose was 1.2 mg/kg. In the dose-expansion cohort, the ORR was 33% and 32% in patients with HER2-positive and HER2-low metastatic BC, respectively. Regarding safety profile, grade 3 or more AEs were observed in 35% of participants^[71]^. The TULIP phase III trial randomized patients with HER2-positive metastatic BC who failed at least two previous lines of treatment to either SYD985 or treatment of physician’s choice. Previous treatment with T-DM1 was allowed. The study met its primary endpoint of PFS. SYD985 significantly prolonged PFS in comparison with control group (7.0 *vs*. 4.9 months, HR: 0.64; *P* = 0.002). However, the final analysis of OS did not show a survival advantage with SYD985 (HR: 0.87; 95% CI, 0.68-1.12; *P* = 0.236) and both arms showed similar ORR. The rate of interstitial lung disease/pneumonitis was 7.6% (22/288 patients), including two grade 5 events (grade 1-2 in 5.2% of patients). To be noted that 21.2% of patients presented grade 3 or more ocular toxicities in the SYD985 group that led to treatment withdrawal in 20.8% of patients^[72]^. The ISPY-P1.01 is a phase I trial evaluating SYD985 in combination with paclitaxel in metastatic BC (NCT04602117).

Trastuzumab rezetecan (SHR-A1811) is a new-generation HER2-directed ADC composed of trastuzumab linked to a novel topoisomerase I inhibitor payload (SHR9265) via a stable and cleavable linker. It has been studied in a phase I first-in-human study including pretreated patients with HER2-mutated/expressing advanced solid tumors. The entire cohort has an ORR of 61.6% (154/250). Interestingly, patients with HER2-positive BC had an ORR of 81.5% (88/108 patients), while patients with HER2-low disease had an ORR of 55.8% (43/77). Moreover, patients with HER2-positive BC and previously treated with T-DM1 had an ORR of 82.4% (14/17). Fifteen patients had previously received an anti-HER2 ADC other than T-DM1, including T-DXd. The ORR was 60% in these patients (9/15)^[73]^. A166 is another HER2-directed ADC composed of humanized HER2-targeting antibody linked to a cytotoxic drug (Duostatin-5, a microtubule inhibitor agent) via a stable cleavable linker. In the cohort of HER2-positive patients, A166 was associated with an ORR of 70%. In addition, median PFS ranged between 9.4 and 12.3 months following the administered doses^[74]^.

Disitamab vedotin (RC48) is a humanized anti-HER2 ADC conjugated with monomethyl auristatin E (MMAE) via a cleavable linker with a bystander effect on cancer cells. The DAR for RC48 is 4.1. In a preclinical model of HER2-positive BC metastatic to the lungs, tumor cells showed resistance to T-DM1 and T-DXd and were moderately sensitive to disitamab vedotin. In the mouse model, the three drugs inhibited the development of lung metastases, and T-DXd and disitamab vedotin were more efficacious than T-DM1^[75]^. Recently, pooled analysis from two phase I trials in patients with HER2-positive and HER2-low metastatic BC demonstrated clinical activity of RC48. The ORR was 40% in the HER2-low group and 43% in the HER2-positive group. The RP2D was 2 mg/kg. The drug presented an acceptable safety profile: decreased neutrophil count, fatigue, and increased GGT were the most frequent grade 3 or more AEs^[76]^. Several phase III trials are currently investigating RC48 in patients with locally advanced or metastatic HER2-positive or HER2-low BC (NCT03500380 and NCT04400695, respectively).

XMT-1522 is another novel HER2-targeting ADC and was compared to T-DM1 *in vitro* and *in vivo* in gastric cancer and BC. Le Joncour *et al*. found that XMT-1522 showed efficacy in BC and gastric cancer cells expressing HER2 and xenograft models both resistant to T-DM1^[77]^. Moreover, ARX788 is an anti-HER2 ADC consisting of anti-HER2 mAb conjugated to amberstatin (AS269) cytotoxic payload. *In vitro* and *in vivo* experience showed that ARX788 is efficacious in models of breast and gastric cancers expressing HER2, including models with acquired resistance to T-DM1^[78,79]^. In the phase I trial, ARX788 showed activity in 31% of patients with heavily pretreated patients with HER2-positive metastatic BC^[80]^. In the ACE-BREAST-02 phase III trial, ARX788 was compared with capecitabine and lapatinib in patients who were previously treated with trastuzumab and taxane. The study met its primary endpoint of PFS^[81]^.

Bispecific or biparatopic mAbs are a promising solution to overcome or prevent resistance to ADCs. Li *et al.* engineered *in vitro* models of HER2-targeting bivalent biparatopic ADCs that demonstrated efficacy against T-DM1 resistant tumors as well as HER2-low expressing cancers. The benefit is due to the targeting of two non-overlapping epitopes on HER2 inducing HER2 receptor clustering and consequently robust internalization, lysosomal trafficking, and degradation^[82]^. Moreover, Zanidatamab zodovotin (ZW49) is a bispecific ADC targeting two non-overlapping HER2 antigens at the same time and linked to an auristatin payload via a C linker. Its DAR is 2. It has been evaluated in HER2-positive solid tumors in a phase I dose-escalation trial. Two dose-limiting toxicities have been reported at the dosage of 1.75 and 2.5 mg/kg. It should be noted that treatment-related keratitis was observed in 43% of patients. The DCR in the cohort of 2.5 mg/kg was 50% in patients with HER2-positive metastatic BC^[83]^. BIO-201 is a novel bispecific ADC that targets TROP-2 and HER2 conjugated with a topoisomerase I inhibitor via a cleavable linker. Preclinical data suggest that BIO-201 is effective in different types of cancer co-expressing TROP-2/HER2 or either of the targets^[84]^.

As previously mentioned, HER3 overexpression can lead to resistance to anti-HER2 drugs. HER3 inhibition using HER3-targeting ADC such as patritumab deruxtecan could be an interesting area to investigate. Patritumab deruxtecan (HER3-DXd) is a HER3-targeting ADC coupled to TOPO1 inhibitor via a C linker. The DAR is 8. It has been evaluated in the U31402-A-J101 phase I/II trial in patients with HER3-expressing metastatic BC. Interestingly, in the subgroup of patients with HER2-positive BC (*n* = 14), HER3-DXd was associated with remarkable clinical activity with ORR of 42.9%, DCR of 92.9%, and median PFS of 11.0 months^[85]^. Zenocutuzumab is a bispecific antibody targeting HER2 and HER3 simultaneously. It has been evaluated in patients with cancers harboring NRG1 fusion. In a phase II trial, responses to zenocutuzumab were observed in 34% of patients (35% in patients with NSCLC, 39% in patients with pancreatic adenocarcinoma, and 50% in patients with BC)^[86]^.

Another strategy to overcome resistance to ADC and increase sensitivity to these agents is the development of non-internalizing ADCs that could target the tumor microenvironment (TME). In fact, the TME, mainly composed of extracellular matrix, abnormal blood vessels and stromal cells such as cancer-associated fibroblasts, plays a crucial role in the growth of tumor cells, neoangiogenesis, and the removal of metabolic waste^[87]^. The TME is associated with some antigens that include enzymes, transcription factors, and checkpoint molecules^[88]^. Several novel ADCs are under clinical development and evaluation in hematological malignancies and solid tumors targeting CD74, CCR7, and CD276^[89,90]^.

### Combination strategies with ADC

Multiple combinations are currently under investigation to overcome acquired resistance to HER2-targeting ADCs and improve the outcomes after failure of these drugs. The most evaluated and promising associations are ADCs with TKI, ICIs, and DNA-damaging agents such as PolyADP-Ribose Polymerase-1 (PARP) inhibitors. The HER2CLIMB-02 is the first phase III study to compare tucatinib combined with T-DM1 *vs*. T-DM1 alone in patients with unresectable HER2-positive BC who received a combination of taxanes with trastuzumab. The results were presented at the 2023 San Antonio Breast Cancer Symposium, and the trial met its primary endpoint of PFS. The combination significantly prolonged PFS in comparison with T-DM1 alone (9.5 *vs*. 7.4 months, HR 0.74). To be noted that 44.1% of patients enrolled in the study had brain metastases at baseline^[91]^. The major limitation of this trial is that it does not compare the combination with T-DXd that is currently the standard of care as a second-line treatment. Moreover, the positioning of this combination is not very clear. Although the results of trials should not be compared, T-DXd was associated with a median PFS of 28.8 months in second line, 17.8 months in T-DM1 refractory patients *vs*. 9.5 months in the HER2CLIMB02. The HER2CLIMB-04 is an ongoing phase II trial evaluating tucatinib in combination with T-DXd in patients with HER2-positive metastatic BC (NCT04539938).

Regarding the combination with ICIs, the KATE2 phase II randomized trial was the first study to evaluate the combination of T-DM1 with atezolizumab, a programmed death ligand-1 (PD-L1), in patients with previously treated HER2-positive metastatic BC. The trial did not meet its primary endpoint of PFS in the intention-to-treat population (8.2 *vs*. 6.8 months; HR: 0.82, 95%CI: 0.55-1.23; *P* = 0.33), showing that the addition of atezolizumab to T-DM1 did not improve PFS and was associated with more adverse events^[92]^. The subsequent KATE3, randomized phase III trial is comparing T-DM1 in combination with atezolizumab *vs*. T-DM1 alone in patients with previously treated HER2-positive and PD-L1-positive metastatic BC (NCT04740918)^[93]^. Moreover, the ASTEFANIA phase III trial is also evaluating T-DM1 and atezolizumab or placebo in patients with HER2-positive BC at a high risk of recurrence following neoadjuvant chemotherapy (NCT04873362)^[94]^. The combination of T-DXd with nivolumab has also been evaluated in a phase 1b trial in patients with HER2-positive or HER2-low metastatic BC. In the HER2-positive metastatic BC, the combination was associated with antitumor activity consistent with prior results of T-DXd alone, raising the question of the utility of adding ICIs to ADCs, which requires more follow-up and additional studies^[95]^.

## CONCLUSION

As previously mentioned, the HER2-targeting landscape has dramatically changed the treatment landscape of patients with HER2-positive BC as well as HER2-low metastatic BC. To date, two drugs are approved by the FDA and EMA for patients with HER2-expressing breast cancer. T-DM1 is the first approved ADC and is currently indicated as adjuvant treatment in patients with HER2-positive BC with residual disease following neo-adjuvant trastuzumab-base chemotherapy. T-DXd is the second HER2-targeting approved ADC in HER2-positive and HER2-low metastatic BC. It defeated T-DM1 in a head-to-head comparison in second-line settings in patients with HER2-positive metastatic BC and sparked the debate on the efficacy of T-DM1 as monotherapy in subsequent therapeutic lines. The impressive clinical efficacy of T-DXd is counterbalanced by the emergence of resistance to this agent and the absence of clinical data and standardized consensus for the treatment of patients after progression on T-DXd. The preferred available regimens could be T-DM1 or the combination of capecitabine, tucatinib, and trastuzumab following the results of the HER2CLIMB phase III trial that showed statistically longer median OS in comparison with capecitabine and trastuzumab^[96]^. The combination of T-DM1 and tucatinib could be another option. Moreover, ongoing clinical trials are assessing T-DXd as a potential first-line treatment, comparing it with the current standard of care and exploring its use in the adjuvant setting. If approved for these indications, it will further complicate the treatment landscape for patients with HER2-positive breast cancer. Investigating next-generation ADCs is crucial, with a need for more efforts to elucidate their mechanisms of action, pharmacodynamic and pharmacokinetic profiles, and the molecular drivers of resistance, which remain poorly understood. Another pertinent concern is whether an ADC can effectively reverse the resistance to another ADC. While this has been demonstrated with T-DXd following T-DM1, it requires confirmation with next-generation ADCs. Combination strategies also offer promise in overcoming resistance, supported by encouraging available data.

## DECLARATIONS

### Copyright

© The Author(s) 2024.
